# Retinal Capillary Ischemia Following Migraine: A Case Report

**DOI:** 10.7759/cureus.75428

**Published:** 2024-12-09

**Authors:** Ana Faria Pereira, Rita Teixeira Martins, Madalena Pinto, Olinda Faria, Susana Penas

**Affiliations:** 1 Department of Ophthalmology, Unidade Local de Saúde de São João, Porto, PRT; 2 Department of Neurology, Unidade Local de Saúde de São João, Porto, PRT; 3 Department of Sense Organs, Faculty of Medicine, University of Porto, Porto, PRT

**Keywords:** migraine, migraine disorder, multimodal, retinal ischemia, visual aura

## Abstract

Migraine, a neurological disorder often accompanied by symptoms such as visual disturbances, nausea, and photophobia, involves complex interactions between genetic and environmental factors, while vascular factors are also implicated, influenced by both genetic predisposition and environmental triggers. This case report discusses a 41-year-old male with a history of migraine with visual aura, presenting with sudden left-eye visual loss. Comprehensive ophthalmologic examination revealed a central scotoma, while multimodal imaging, including spectral-domain optical coherence tomography (SD-OCT), showed focal alterations in the outer plexiform layer. Despite a normal fundoscopic exam and head CT, the scotoma persisted for two weeks before resolving spontaneously. Follow-up imaging and visual field tests showed complete resolution of abnormalities. This case underlines the potential for retinal microvascular alterations in migraine, which may predispose patients to retinal ischemia, necessitating thorough eye and neurological assessments in such cases. Conditions like acute macular neuroretinopathy (AMN) and paracentral acute middle maculopathy (PAMM) may share a common pathophysiology with migraine.

## Introduction

Migraine is a neurological disorder characterized by recurrent headaches, often accompanied by symptoms such as visual disturbances, nausea, and light sensitivity. Migraine with typical aura is highly prevalent, affecting 8% of the general population. Typical migraine aura (MA) symptoms are reversible visual, sensory, or language disturbances, but visual aura symptoms are the most common, occurring in 98-99% of MAs. Visual aura frequently presents with symptoms of flashes of bright light, “foggy” vision, zigzag lines, and scotoma. In some cases, persistent monocular visual loss and abnormal ophthalmological findings have been reported [1].

Ocular migraine and migraine with visual aura are related but distinct conditions, each with unique pathophysiological mechanisms and clinical presentations. Ocular migraine, known as retinal migraine, is characterized by transient visual disturbances or vision loss in one eye, typically caused by vasospasm or transient reduction in the retina or optic nerve blood flow, resulting in temporary ischemia. These vascular changes lead to temporary vision impairment, emphasizing the critical role of blood flow in retinal function. In contrast, migraine with visual aura involves visual disturbances that precede or accompany a migraine headache, affecting both visual hemifields [2,3]. These disturbances are attributed to cortical spreading depression, a wave of neuronal and glial depolarization propagating across the visual cortex. This condition also involves the trigeminovascular system, where activation of the trigeminal nerve leads to the release of neuropeptides that cause inflammation and further vascular changes [2,4]. Ocular migraine may not be followed by a headache, unlike migraine with visual aura, which typically leads to a throbbing headache. Understanding these pathophysiological differences, including the role of vascular changes and vasospasm, is crucial for accurate diagnosis and effective management of these variants. Whilst exact mechanisms behind migraine remain elusive, emerging research has been unveiling the connection between this mechanism and retinal capillary ischemia, emphasizing the intricate interplay between ocular and nervous system vascular beds [3].

MA presents a notable risk factor for severe cardiovascular disorders, including ischemic and hemorrhagic stroke, myocardial infarction, atrial fibrillation, and perioperative stroke. The employment of combined oral contraceptives amplifies the likelihood of vascular incidents by up to 13 times, a significant concern given that a substantial proportion of migraine sufferers are women in their reproductive years. Furthermore, the differential diagnosis includes cerebrovascular disorders, epilepsy, and other life-threatening neurological conditions. Within clinical contexts, distinguishing MA from transient ischemic attacks and stroke poses considerable challenges. Migraine ranks as the third most prevalent stroke imitator, trailing behind seizures and psychiatric disorders, contributing to 18% of erroneously administered thrombolytic treatments [5-8]. Conversely, individuals with undetected strokes in an emergency department frequently initially receive a misdiagnosis of "migraine". Hence, an accurate diagnosis of MA is paramount to distinguish it from other potentially life-threatening conditions [1,6].

Enhancing our understanding of MA holds the utmost importance for refining diagnostic accuracy and preventing inappropriate therapies like IV thrombolysis, as well as unnecessary work-ups and tertiary prevention measures for cerebrovascular diseases [6,7].

## Case presentation

A 41-year-old male presented to the ophthalmology emergency department at Hospital de São João, Porto, Portugal, with complaints of sustained left eye (OS) visual loss for the past day. He reported having had a unilateral, pulsatile headache with an intensity of 6/10 the previous day, accompanied by motion-aggravated pain, nausea, and light sensitivity. This headache episode was preceded by his usual migraine symptoms, including flashing lights and brief periods of transient vision loss.

**Figure 1:**
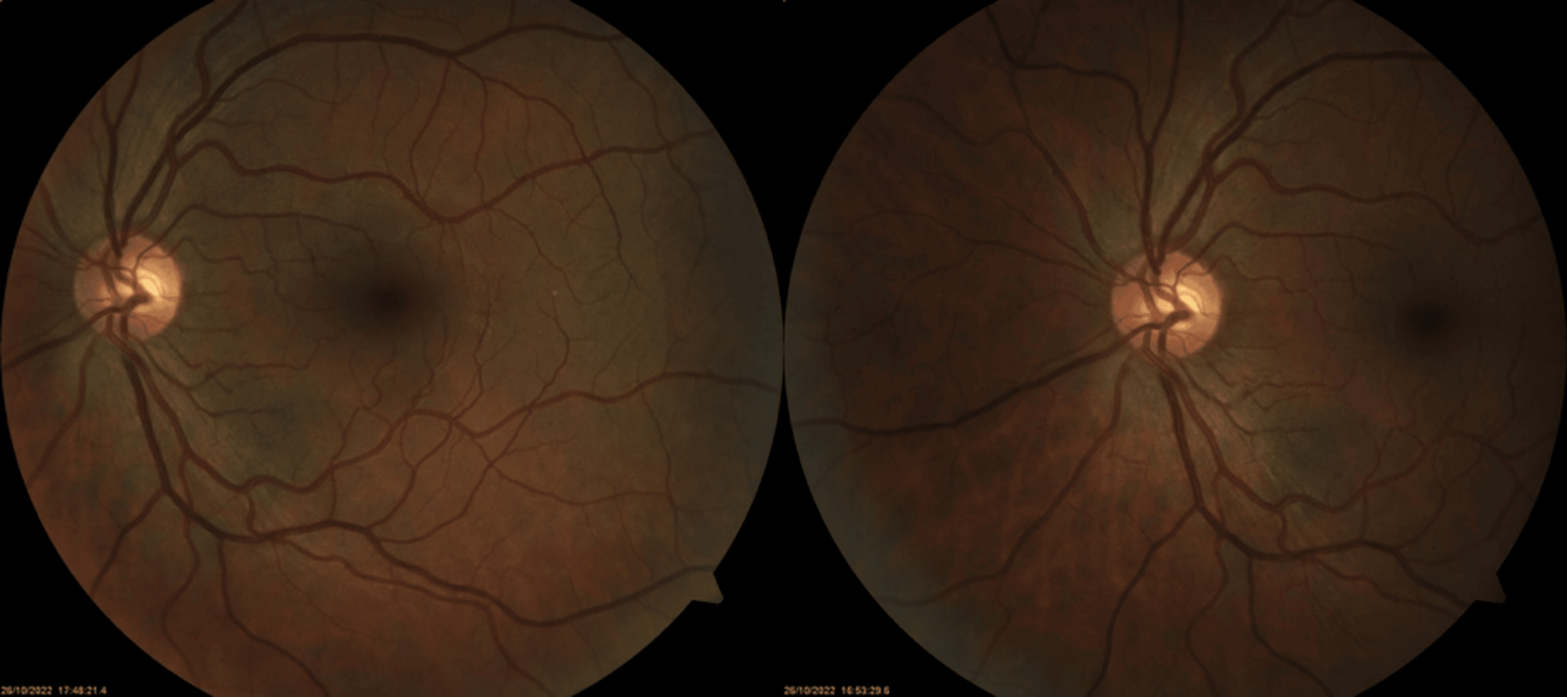
Color fundus photographs showing no abnormalities.

At presentation, his best corrected visual acuity (BCVA) was 20/20 in the right eye (OD) and 20/40 OS. Anterior segment examination was unremarkable in both eyes and intra-ocular pressure was 12 mmHg in both eyes (OU). Fundoscopic examination OU was unremarkable, with normal optic discs, macula, and retinal vessels, with no signs of vascular occlusions or emboli (Figure 1). A neurologic examination carried out by a neurologist yielded no notable findings, and the head CT scan was normal.

His medical history included migraines, with no known ophthalmic conditions, no history of eye surgery, and no other ocular issues. He denied using medications or other drugs, as well as energy drinks, and reported no history of smoking. He had been suffering from migraine with visual aura since childhood and referred to having his typical symptoms in this last episode. The pain is typically alleviated with oral intake of naproxen. However, he reported persistent vision loss OS, deviating from its typical pattern by lasting for 24 hours.

**Figure 2:**
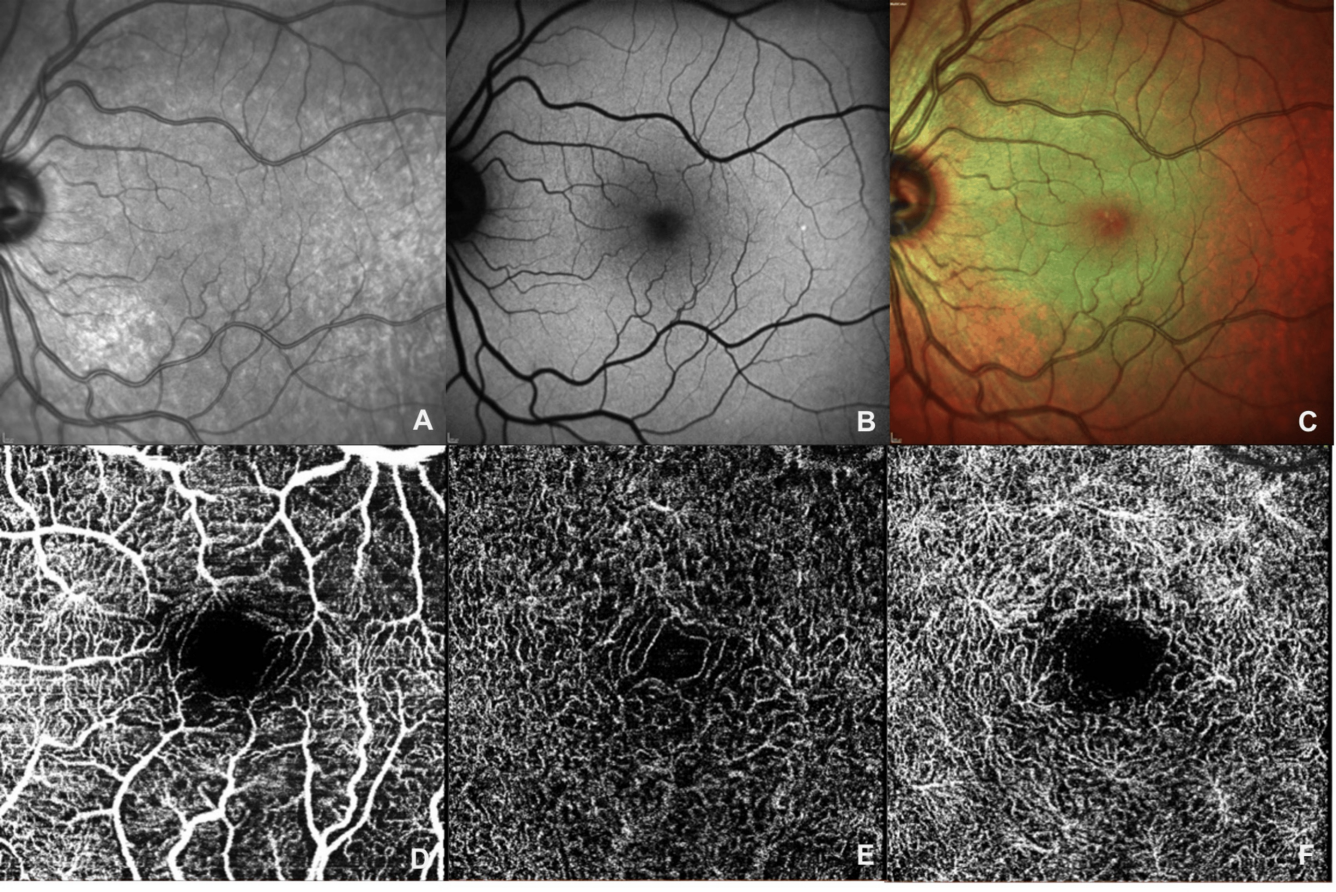
Near-infrared imaging (A), fundus autofluorescence (B), multicolor imaging (C), and optical coherence tomography angiography (OCT-A) of the left eye (OS) at the initial visit, including the superficial (D), intermediate (E), and deep capillary plexuses (F), reveal no abnormalities.

**Figure 3:**
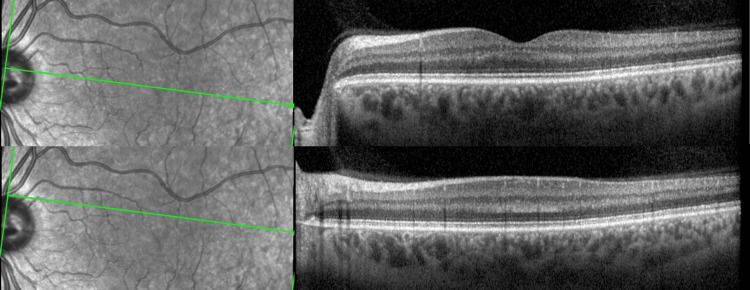
SD-OCT images showing focal increased reflectivity of the outer plexiform layer (OPL) in the parafoveal region. SD-OCT: spectral-domain optical coherence tomography

Multimodal imaging with spectral-domain optical coherence tomography (SD-OCT, Spectralis HRA-OCT, Heidelberg), fundus auto-fluorescence (FAF), near-infrared (NIR), multicolor and optical coherence tomography angiography (OCT-A) was performed. FAF, NIR, and OCT-A imaging were unremarkable in both eyes (Figure 2), but SD-OCT revealed focal areas of increased reflectivity in the outer plexiform layer (OPL) within the nasal and temporal parafoveal regions of the OS (Figure 3). Peripapillary retinal nerve fiber layer thickness was normal.

**Figure 4:**
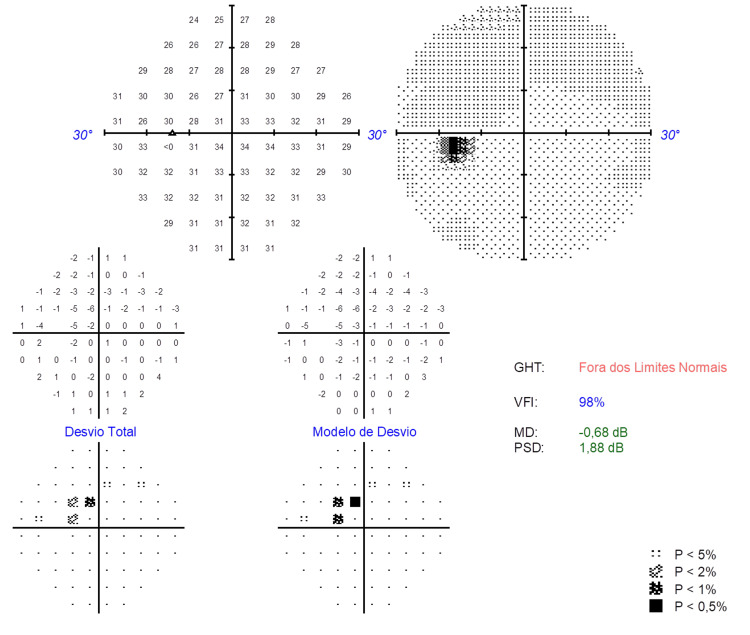
The 30-2 Humphrey visual field test (reliable) showing a central scotoma in the left eye (OS) (it was normal in the right eye (OD)).

A 30-2 Humphrey visual field (VF) test confirmed a central scotoma in the OS, corresponding to the affected area on the SD-OCT (Figure 4), and the VF in the OD was normal.

The scotoma persisted for two weeks before resolving spontaneously. At the subsequent one-month follow-up appointment, the patient had no ongoing complaints or symptoms, and both SD-OCT and VF test abnormalities had entirely resolved, with no structural sequelae in the outer retinal layers.

## Discussion

The sudden onset of a central scotoma accompanied by a history of migraines warrants assessment for potential acute retinal ischemia. We suggest that in some cases there may be alterations in retinal microvasculature in migraine potentially raising vulnerability to retinal ischemia [5].

The spectrum of retinal ischemia includes a variety of manifestations, extending from microvascular alterations as observed in this case, to macrovascular lesions such as branch and central retinal artery occlusions. Conditions such as acute macular neuroretinopathy (AMN) and paracentral acute middle maculopathy (PAMM), known to involve the intermediate and deep vascular capillary layers, respectively, have been observed in patients with migraines, suggesting a potential shared underlying pathophysiology. Despite the absence of typical fundoscopic, NIR, auto-fluorescence, and OCT-A features for AMN or PAMM in this case, the OCT and perimetric findings are consistent with deep capillary ischemia [9].

Deep capillary ischemia, PAMM, retinal arterial occlusion, and cerebral infarction share the same risk factors, such as hypertension, smoking, heart disease, diabetes, obesity, migraines, dyslipidemia, and female gender. Hence, clinicians may consider deep capillary retinal ischemia as an early sign of retinal arterial occlusions. Notably, migraines with aura pose a greater risk of retinal arterial occlusion than migraines without aura, necessitating prompt neurovascular evaluation, as performed in the reported case [9].

Clinicians should also explore alternative causes of acute retinal ischemia, including giant cell arteritis (GCA), thrombosis, hypercoagulability, and collagen disorders. In older patients (over 55 years old) with compatible symptoms, ruling out GCA is crucial. However, given our patient's age, clinics, and absence of other suggestive symptoms, we deemed GCA unlikely and refrained from ordering inflammatory markers. Nonetheless, clinicians must carefully manage isolated acute retinal ischemia, ruling out acute stroke and GCA, especially if the patient presents within 24 hours of vision loss [2].

Microvascular assessment using OCT and OCT-A is vital to detect subclinical vascular abnormalities, especially in cases with no fundoscopic visible lesions [3]. While typical MA usually resolves within an hour, our patient's prolonged episode likely triggered the retinal ischemic cascade through two mechanisms: spreading depression of retinal neurons and vasospasm within ciliary or retinal blood vessels, leading to sublethal hypoxia, blood flow obstruction, oxidative stress, and cell death. This phenomenon, causing interrupted flow in the deep retinal vascular complex, resulted in the observed OPL infarction in our case [2].

Regarding the full clinical, radiological, and VF improvement in our patient, this is due to a cascade of neuroprotective responses typically activated after an ischemic event. This includes the upregulation of survival pathways and the release of endogenous growth factors such as brain-derived neurotrophic factor (BDNF) and glial cell line-derived neurotrophic factor (GDNF). These factors promote neuronal survival, reduce apoptosis, and support synaptic plasticity. Additionally, Müller cells and astrocytes play a crucial role in maintaining the retinal environment by modulating inflammation and promoting clearance of cellular debris. Vascular endothelial growth factor (VEGF) also contributes to the repair process by facilitating angiogenesis, thereby restoring blood flow and oxygen supply to the damaged retina. Collectively, these mechanisms not only help in structural repair of retinal layers but also in functional recovery of visual pathways, leading to improvements in VF and overall visual acuity post-ischemia [10].

Acute retinal ischemia, which may require urgent neurovascular evaluation and GCA assessment, should be considered in patients with migraines experiencing persistent visual changes. Diagnosis of deep capillary ischemia requires high suspicion as it can occur even without significant changes in visual acuity, VF, or fundoscopy [2,9].

This case emphasizes the importance of comprehensive eye and neurological evaluations in individuals with migraines, especially if they report unusual visual symptoms [6]. Diagnostic tools such as OCT, OCT-A, VF, and others should be considered, guided by the urgency of the presentation, available resources, and clinical judgment, with prioritization of accessible tests and close follow-up in emergency settings.

## Conclusions

The sudden onset of a central scotoma in individuals with a history of migraines warrants a thorough assessment for potential acute retinal ischemia. The spectrum of retinal ischemia can range from microvascular to macrovascular lesions. Migraines with aura present a higher risk for retinal arterial occlusion, necessitating prompt neurovascular evaluation. Clinicians should also consider other potential causes of acute retinal ischemia, such as GCA, particularly in older patients. Microvascular assessment using OCT and OCT-A is crucial in detecting subclinical vascular abnormalities, especially in cases without visible fundoscopic lesions.
